# Effects of different rates of propofol with or without S-ketamine on ventricular function in healthy cats – a randomized study

**DOI:** 10.3389/fvets.2023.1272949

**Published:** 2023-12-11

**Authors:** Sabrine Marangoni, Matheus Ubiali, Francieli Ambrosini, Larissa Jahnel, Julia M. Vilani, Paulo V. Steagall, Ricardo Guilherme D’Otaviano de Castro Vilani

**Affiliations:** ^1^Department of Veterinary Medicine, Federal University of Paraná, Juvevê, Curitiba, PR, Brazil; ^2^Department of Veterinary Clinical Sciences and Centre for Companion Animal Health and Welfare, Jockey Club College of Veterinary Medicine and Life Sciences, City University of Hong Kong, Kowloon, Hong Kong SAR, China; ^3^Department of Clinical Sciences, Faculty of Veterinary Medicine, Université de Montréal, Saint-Hyacinthe, QC, Canada

**Keywords:** feline, propofol, procedural sedation, S-ketamine, cardiovascular

## Abstract

Propofol is used for anesthetic induction in cats and procedural sedation in countries where alfaxalone is not available. Studies have reported propofol-related effects in echocardiography variables in dogs and humans. However, there is a lack of echocardiography studies investigating propofol-related effects on cats. This study aimed to use echocardiography to investigate echocardiographic changes in three protocols using propofol: propofol-slow (2 mg/kg/min, PS); propofol-fast (8 mg/kg/min, PF); propofol-ketamine (S-ketamine 2 mg/kg bolus followed by propofol 2 mg/kg/min; PK) in healthy premedicated (gabapentin–buprenorphine–acepromazine; 200 mg/cat, 0.4, and 0.1 mg/kg, respectively), non-intubated cats. Echocardiographic measurements were obtained at three time points: baseline (before the administration of propofol), end of propofol titration (end-point, T0), and 15 min after T0 (T15). Propofol at a lower rate continued from T0 to T15. Echocardiographic and physiological variables included fractional shortening (FS%), ejection fraction (EF%), HR, BP, and others. Propofol requirements at T0 for PF, PS, and PK groups were 5.0 ± 0.9, 3.8 ± 0.7, and 2.4 ± 0.5 mg/kg, respectively. EF% neither change over time nor between groups. PF and PK showed a reduction in FS% at T0 (47 ± 6 to 34 ± 6 and 42 ± 6 to 36 ± 5, respectively). BP reduced significantly in PF and PS groups (136 ± 26 to 105 ± 13 and 137 ± 22 to 115 ± 15 mmHg, respectively). It is unclear whether changes in echocardiography variables were of clinical relevance related to treatment groups or a result of within-group individual responses.

## 1 Introduction

Propofol is a hypnotic drug used for induction and maintenance of anesthesia in veterinary medicine. The drug is also used for small animal procedural sedation during short-term non-invasive procedures (e.g., diagnostic procedures, nasoesophageal feeding tube placement, bandage changes, and wound cleaning) (1). The use of propofol for procedural sedation results in the activation of the inhibitory gamma-aminobutyric acid (GABAA) receptors, promoting unconsciousness with loss of protective reflexes while decreasing stroke volume and thereby reducing cardiac output (CO) in a dose-dependent manner (2–5). Propofol also decreases heart rate (HR), myocardial contractility, and arterial blood pressure in cats and humans (6, 7). Decreases in blood pressure and the direct negative inotropic effects are closely related to plasma concentrations of propofol (8). Additionally, propofol causes hypoventilation, hypercapnia, and hypoxemia that may lead to other secondary hemodynamic changes and sympathetic activation (9). These changes may be influenced by both dose and speed of the administration (8, 9). Ketamine, a dissociative anesthetic commonly used in many species, is available as a racemic mixture or S-ketamine. S-ketamine is often mentioned as twice as potent as racemic ketamine with improved antihyperalgesic effects and lower adverse effects (1). Indeed, some authors suggest that a slower rate of administration and/or the use the propofol in combination with ketamine (to decrease the propofol dosage) may reduce propofol-related adverse cardiovascular effects (9–11). In healthy dogs, fast administration of propofol reduced arterial blood pressure while cardiac output was sustained by compensatory chronotropic response (8). However, conclusive evidence to these benefits in cats is lacking. Clinical experience shows that it is not uncommon to observe cardiorespiratory depression with the administration of propofol for procedural sedation in cats. A “quick sedation” may be detrimental for the patient, especially when intubation is not performed and hypoventilation is present in cats with suboptimal anesthetic monitoring (1).

Cardiac systolic function depends on several factors such as preload and afterload, myocardial contractility, distensibility (contraction and relaxation), rhythm, and heart rate, which are all ultimately affected by age, drug administration, disease states, and circulating volume (12). To ensure safe sedation or anesthesia, it is essential to assess the influence of anesthetics on these variables including propofol. Echocardiography has become an important diagnostic technique for detecting hemodynamic changes related to induction of anesthesia with propofol in dogs (8, 13) and procedural sedation injectable protocols in cats (14, 15). However, there is a lack of echocardiography studies investigating the effects of propofol administered at different rates in cats and particularly mimicking procedural sedation.

This study aimed to use transthoracic echocardiography to investigate the echocardiographic changes and its potential hemodynamic effects in some cardiovascular variables (e.g., non-invasive blood pressure and HR) of three protocols using propofol in healthy premedicated, non-intubated cats. The hypothesis was that the administration of propofol at a fast infusion rate would result in greater adverse left ventricular systolic changes than a slow infusion rate with or without S-ketamine in these cats.

## 2 Materials and methods

### 2.1 Animals

This study is approved by the ethics committee (no. 024/021) and is reported according to the CONSORT guidelines.^1^ The experimental study was performed at the veterinary teaching hospital of the Federal University of Parana (UFPR, Curitiba, Brazil) from April to July 2021.

A total of 24 domestic shorthair healthy male cats from a single animal shelter, scheduled for orchiectomy, were enrolled in a prospective, randomized, masked, experimental trial after receiving written consent. At the end of the study, cats were returned to the shelters for adoption. Sample size estimates were derived from power calculations using G*Power^2^ based on median and effect sizes with the aim to achieve 80% power and alpha-error rate of 0.05 (two-sided) obtained from the results (i.e., FS% used as outcome of interest) after the first eight anesthetic episodes (i.e., three for two groups and two for one group).

Inclusion criteria included healthy male cats of any breed, > 2 kg body weight, > 1 year of age, and American Society of Anesthesiology (ASA) status I. Cats were deemed healthy based on history, physical examination, a complete blood count (CBC), serum chemistry profile (glucose, blood urea nitrogen, creatinine, serum total protein, serum alkaline phosphatase, alanine aminotransferase, and aspartate aminotransferase) within reference values, and the absence of abnormal cardiac sounds (e.g., gallop sound) or murmurs during thoracic auscultation. Exclusion criteria included feral behaviors (i.e., that would impact cat handling), obesity (body condition score > 7 on a scale 1–9), or clinical signs of disease. Cats with suspected structural or functional cardiac abnormalities, including hypertrophic cardiomyopathy and interventricular septal thickness at end-systole and end-diastole (IVSs and IVSd) of ≥6 mm (16), were excluded after baseline measurements.

Animals were admitted and placed in individual cages containing water, food bowl, cardboard boxes, litter box, and blankets and underwent an approximately 12 h of acclimatization period before the study. Cats were housed and handled according to feline-friendly interactive and handling techniques (17). Oral gabapentin (100 mg, Drogavet, PR, Brazil) was administered approximately 12 h and 1 h before the procedure (18), to reduce stress and fearfulness (17, 18). A complete physical examination was performed approximately 90 min after the first dose of gabapentin, and hair was clipped on the right thoracic limb (for venous access), left pelvic limb above the metatarsal pad (for non-invasive blood pressure), and on both sides of the thorax (for transthoracic echocardiography; TTE). Food and water were withheld for 6 and 2 h, respectively, before premedication (see below). The second dose of gabapentin was administered after the removal of water.

### 2.2 Groups and procedures

All cats were premedicated with butorphanol (0.4 mg/kg; Torbugesic, 10 mg mL; Pfizer, United States) and acepromazine (0.1 mg/kg, Acepran 0.2%, Vetnil, Brazil) mixed in the same syringe and administered intramuscularly into the epaxial lumbar muscles. After 30 min, the cats were gently positioned in right lateral recumbency and wrapped in a towel for placement of an over-the-needle catheter in the right cephalic vein. A single investigator (SM) was responsible to interact with the cats (i.e., handling and housing, gabapentin administration, premedication, and venous catheter placement), to mitigate fear–anxiety–stress response related to unpredictable interactions.

Standardized measurements of TTE were made using a consistent technique so that the same echocardiographic images were obtained using the same sequence of events. All variables were measured according to the recommendations of the American College of Veterinary Internal Medicine (19). Transthoracic echocardiography measurements were recorded at three time points: baseline (immediately before the administration of propofol), immediately at the end of propofol titration (T0), and 15 min after T0 (T15). A veterinarian with experience in cardiology (FA) performed all measurements using a 12 MHz sector transducer attached to a US machine (Affiniti 50, Philips, WA, USA). Physiological variables recorded were respiratory rate (FR) by observation of thoracic excursions, peripheral capillary oxygen saturation (SpO_2_), and electrocardiographic (ECG) monitoring using a multiparametric monitor (LifeWindow, Multi-Parameter Physiologic Monitor LW9x, Digicare Animal Health, RJ, Brazil). Non-invasive blood pressure (BP) was measured using a Doppler ultrasound (Doppler 811-B, Parks Medical Electronics, Inc., Oregon, United States). Measurements were collected in triplicate at all time points and recorded at every 5 min. The mean value was then calculated and recorded. A cuff (NIBP cuff, Digicare Animal Health, RJ, UAS) with a width of approximately 40% of limb circumference was placed above the tarsus. Conductive gel (Carbogel ULT, Ind., SP, Brazil) was applied before positioning the flat piezoelectric crystal probe (8.2 MHz) above the metatarsal pad and over the medial plantar artery. HR was measured by ECG monitoring and recorded at the time of CO measurement. Rectal temperature (RT) was measured at T0 and T15 using a digital thermometer. Oxygen was delivered via a modified Mapleson D breathing system connected to a face mask with a fresh gas flow at 400 mL/kg/min throughout the procedure.

All investigators were masked to group assignment except for the individual performing randomization, drug preparation, and administration (JV), who was not involved in the subsequent steps of the study. A 60-ml syringe containing propofol (Propotil 1%, Midpharma, Dongkook Pharm. Co., South Korea) was connected to an infusion pump (SYS3010, Medcaptain Medical Technology Co., China) with its electronic display covered with paper to conceal treatment allocation (i.e., blinding). Each cat was assigned a number (1–24) based on their order of arrival and randomly^3^ divided into one of three treatments: propofol infusion rate of 2 mg/kg/min (propofol slow; group PS); 8 mg/kg/min (propofol fast; group PF); or a single bolus of S-ketamine (2 mg/kg; Ketamin, Cristália, Brazil) injected manually for 15 s followed by 2 mg/kg/min (propofol–ketamine; group PK). Syringes with S-ketamine were diluted with saline 0.9% to a final volume of 0.5 mL. PF and PS groups received 0.5 mL of saline 0.9% before propofol infusion.

After baseline measurements, treatments were administered as above until an end-point (T0) was reached according to one investigator (RV), in order to mimic procedural sedation in the clinical setting. The end-point was considered as follows: the absence of lateral palpebral reflex, reduced consciousness, decreases in jaw tone, and the ability of pulling out the cat’s tongue gently without resistance. Cats were not intubated. However, a cuffed endotracheal tube and a laryngoscope were available in the case of apnea. Once T0 was achieved, the propofol infusion was decreased to 0.4 mg/kg/min in all groups and progressively decreased by 0.1 mg/kg/min every 5 min to maintain a consistent level of sedation within each group. After 15 min (T15), TTE values, HR, FR, and RT were recorded once again. The total amount of propofol required to maintain anesthesia (i.e., propofol administered from T0 to the end of echocardiography in T15) was recorded. Afterward, the cat was prepared for surgery, and orchiectomy was performed by one veterinarian (LJ) using an intratesticular block combined with subcutaneous incisional lidocaine (3 mg/kg; 20 mg/mL, Xylestesin, Cristália, Brazil). For the surgical procedure, propofol infusion rates were administered to provide surgical depth of anesthesia. Meloxicam (0.2 mg/kg; 2 mg/mL, Maxicam, Ourofino, Brazil) was injected subcutaneously after surgery. Fluid therapy was not administered unless the cat was hypotensive with systolic blood pressure lower than 90 mmHg. Anesthetic recovery was monitored, and wet food was offered 1–2 h after anesthetic recovery.

### 2.3 Echocardiography measurements and techniques

Left ventricle (LV) size and systolic function were measured using standard right parasternal short-axis view (PSAx) and long-axis view (PLAx) and a left apical view. Echocardiography variables included LV dimension at end-systole (LVIDs) and end-diastole (LVIDd), at the level of the chordae tendineae from right parasternal short-axis images. Left ventricle ejection fraction (EF%) was determined using Simpson’s method from a right parasternal long-axis view. Fractional shortening (FS% = [LVIDd-LVIDs]/LVIDd × 100%) was calculated. After analyzing the aortic flow from left apical images, CO (L/min) was calculated from aortic velocity spectra using the equation: (CO = VTI x aortic cross-sectional area x HR) (20). In addition, tricuspid annular plane systolic excursion (TAPSE) and mitral annular plane systolic excursion (MAPSE) were evaluated by M-mode from the left apical four-chamber view. Heart rate and BP were measured and recorded (20).

## 3 Statistical analysis

Data were analyzed using R software version 4.1.0 (https://www.r-project.org; *dplyr*, *rstatix*, *reshep*, *stats*, *PMCMRplus*, and *ggplot2* packages). The mean of triplicate measurements was calculated and used for echocardiography outcomes during data analysis. Descriptive statistical analysis was performed with mean, median, standard deviation, and 25 and 75% percentile of quantitative variables, according to time points and groups. Data distribution was analyzed using the Shapiro–Wilk test. For the variables with normal distribution, the difference between the groups was analyzed using ANOVA followed by post-hoc Tukey’s test (LVIDd, LVIDs, and BP). For data without normal distribution, groups were compared using the Kruskal–Wallis test followed by Dunn’s test (CO, EF% Simpson, HR, SpO_2_, MAPSE, and TAPSE). Temporal changes were analyzed using repeated measures ANOVA followed by paired t-test with Bonferroni correction for parametric variables or the Friedman test followed by the Nemenyi test for non-parametric variables. The analyses were considered significant when *p <* 0.05.

## 4 Results

**Figure 1:**
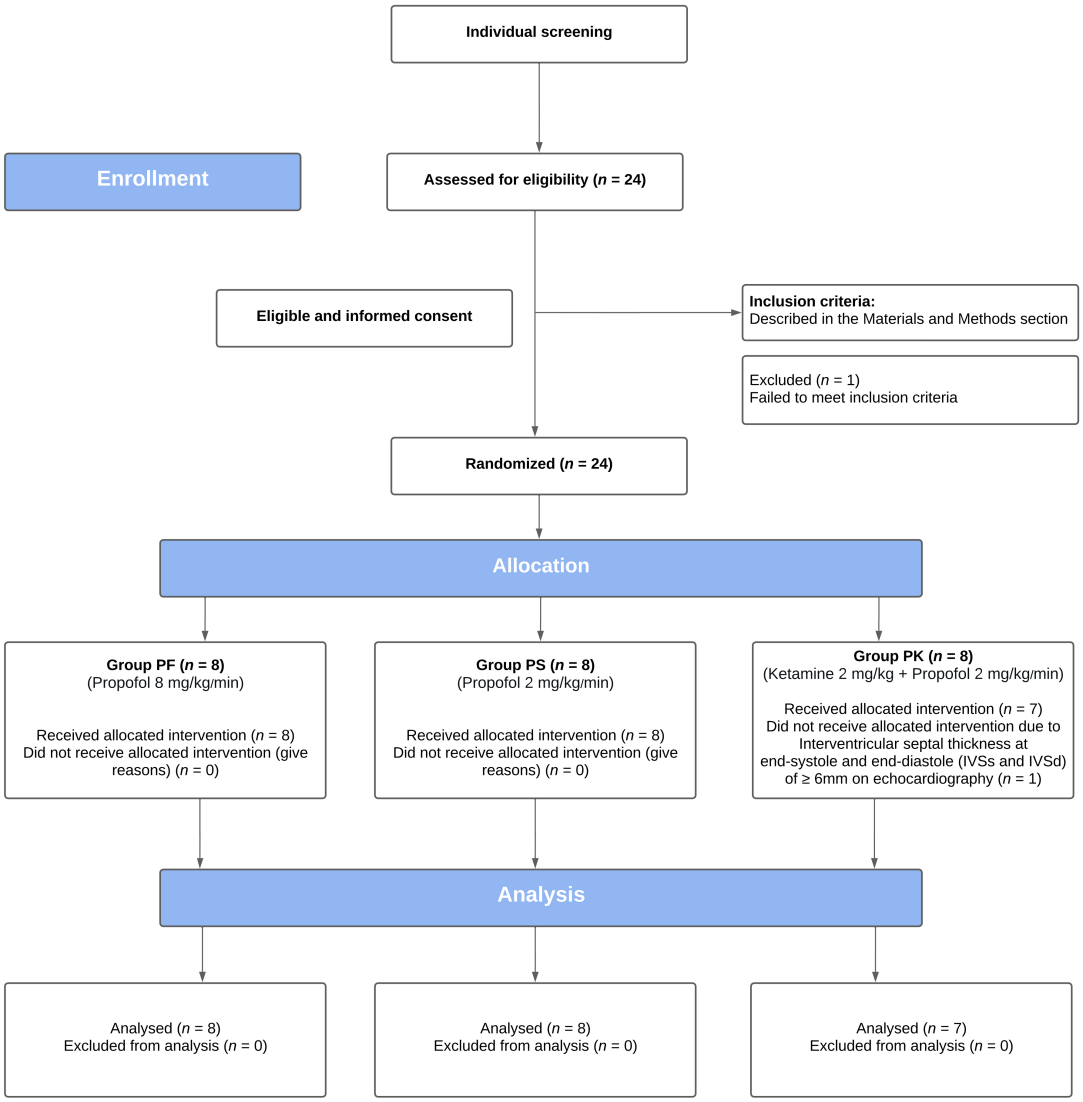
CONSORT flow diagram of a prospective, blinded, randomized, clinical trial, comparing the echocardiographic effects of three protocols for procedural sedation premedicated with gabapentin-butorphanol-acepromazine (200 mg/cat, 0.4 and 0.1 mg/kg, respectively; baseline). Cats were randomly assigned to three groups (PF, 8 mg/kg/min; PS, 2 mg/kg/min; and PK, S-ketamine 2 mg/kg followed by propofol 2 mg/kg/min).

Of the 24 cats enrolled, 23 were included in the study (Figure 1). One cat was excluded from the group PK because of evidence of asymptomatic hypertrophic cardiomyopathy identified at the time of the first echocardiography (Table 1).

**Table 1:** Demographic information of 23 male domestic shorthair cats enrolled in a study comparing the echocardiographic effects of three protocols for procedural sedation premedicated with gabapentin–butorphanol–acepromazine (200 mg/cat, 0.4 and 0.1 mg/kg, respectively; baseline).

Demographic	PF	PS	PK
Cats (n)	8	8	7
Body weight (kg)	3.5 ± 0.4	3.9 ± 0.4	3.6 ± 1.0
Age (years)	2.0 ± 2.1	2.1 ± 2.2	2.3 ± 1.8
Body condition score (1–9)	5 ± 2	5 ± 2	4 ± 2

Cats were assigned to three protocols using propofol: group PF (propofol infusion 8 mg/kg/min); group PS (propofol infusion 2 mg/kg/min); and PK (S-ketamine 2 mg/kg and propofol infusion 2 mg/kg/min).

Significant within-group changes were observed for FS%, HR, and BP in PF; for LVIDd, FS%, CO, and HR in PK; and for HR and BP in PS. There was no statistically significant difference in MAPSE/TAPSE within groups at any time points. No significant differences in treatment comparisons for echocardiography variables were observed at any time point. Although propofol requirements at T0 were significantly different among groups, there was no difference in the total amount of propofol infusion required to maintain anesthesia. Inferential analyses for various variables are presented in Tables 2, 3. Apnea was not observed during the study. Two cats had FS% near to or lower than published reference values (28 to 62%) (21, 22): one cat in PF (i.e., 26%) and another one in PK (i.e., 29%) at T0. The FS% values were returned to normal values in all individuals at T15. However, PF caused bradycardia (< 100 bpm) (23) in five cats. All cats recovered uneventfully from surgery.

**Table 2:** Echocardiographic variables of healthy male cats premedicated with gabapentin–butorphanol–acepromazine (200 mg/cat, 0.4 and 0.1 mg/kg, respectively; baseline) at the end-point (T0) and after 15 min of end-point (T15).

Variable	*p*-value of for group comparisons	Groups	Baseline	T0	T15	*p-value between time points*
LVIDd (cm)		PF	1.52 ± 0.22	1.46 ± 0.20	1.44 ± 0.23	0.666
		PS	1.52 ± 0.28	1.51 ± 0.14	1.53 ± 0.20	0.962
		PK	1.53 ± 0.16	1.40 ± 0.15	1.57 ± 0.19	0.035*^b^
	*p*		0.967	0.477	0.231	
LVIDs (cm)		PF	0.80 ± 0.18	0.96 ± 0.14	0.82 ± 0.13	0.11
		PS	0.81 ± 0.16	0.94 ± 0.11	0.85 ± 0.19	0.101
		PK	0.87 ± 0.10	0.89 ± 0.07	0.91 ± 0.07	0.639
	*p*		0.35	0.289	0.235	
FS%		PF	47.9 ± 6.1	34.4 ± 6.2	42.9 ± 2.3	<0.001*^a,b^
		PS	45.2 ± 4.9	37.3 ± 5.3	43.0 ± 9.5	0.113
		PK	42.6 ± 6.0	36.1 ± 5.0	41.5 ± 6.3	0.035*^a,b^
	*p*		0.81	0.544	0.751	
TAPSE (mm)		PF	9.5 (7.9–11.0)	8.3 (7.2–10.5)	8.8 (8.7–9.8)	0.223
		PS	8.5 (7.2–10.5)	8.5 (7.9–10.8)	9.8 (8.9–10.2)	0.607
		PK	8.7 (7.9–10.6)	8.3 (6.9–10.8)	10.1 (8.0–12.5)	0.156
	*p*	PF	0.884	0.62	0.686	
MAPSE (mm)		PF	5.8 (5.0–8.5)	5.5 (5.0–6.7)	6.1 (4.8–7.2)	0.908
		PS	5.9 (5.5–7.0)	5.6 (5.5–7.0)	6.5 (5.9–8.0)	0.093
		PK	5.6 (4.4–7.5)	5.2 (3.9–5.7)	5.1 (4.7–5.8)	0.368
	*p*		0.891	0.503	0.527	
CO (L/min)		PF	0.40 (0.38–0.45)	0.40 (0.39–0.40)	0.35 (0.30–0.40)	0.505
		PS	0.40 (0.30–0.50)	0.35 (0.27–0.40)	0.30 (0.27–0.32)	0.068
		PK	0.40 (0.30–0.50)	0.30 (0.25–0.35)	0.30 (0.25–0.30)	0.025*^c^
	*p*		0.891	0.503	0.527	
EF% Simpson		PF	50.6 (47.4–57.5)	48.4 (45.9–56.7)	48.2 (45.5–58.2)	0.687
		PS	51.7 (44.7–55.4)	39.8 (36.5–48.8)	47.6 (44.0–52.3)	0.205
		PK	48.6 (43.2–49.9)	48.8 (42.0–51.0)	50.8 (47.4–51.5)	0.367
	*p*		0.784	0.834	0.243	

Anesthesia was induced with intravenous (IV) propofol 8 mg/kg/min (propofol fast; PF, *n* = 8) or propofol 2 mg/kg/min (propofol slow; PS, *n* = 8) or a single bolus of S-ketamine (2 mg/kg) followed by propofol 2 mg/kg/min (propofol–ketamine; group PK; *n* = 7). Data are presented as either mean ± standard deviation or median (interquartile range).

CO (L/min), cardiac output measured from aortic velocity; EF% Simpson, ejection fraction; FS%, fractional shortening; LVIDd, left ventricular internal diameter in diastole; LVIDs, left ventricular internal diameter in systole; MAPSE, mitral annular plane systolic excursion; TAPSE, tricuspid annular plane systolic excursion. *Significant difference between time points (*p* < 0.05). aSignificant differences between baseline x T0; bSignificant differences between T0 x T15; cSignificant differences between baseline x T15.

**Table 3:** Heart rate (HR), non-invasive blood pressure (BP), and propofol requirements of healthy male cats premedicated with gabapentin–butorphanol–acepromazine (200 mg/cat, 0.4 and 0.1 mg/kg, respectively; baseline), at the end-point (T0) and after 15 min of the end-point (T15).

Variable	*p*-value for group comparisons	Groups	Baseline	T0	T15	*p*-value between time points
HR (beats min)		PF	154 (134–193)	124 (92–149)	88 (81–128)	0.011*^c^
		PS	121 (112–138)	102 (90–113)	89 (84–97)	0.006*^c^
		PK	136 (111–159)	106 (83–126)	85 (81–122)	0.004*^a,c^
	*p*		0.967	0.477	0.231	N/A
BP (mmHg)		PF	136 ± 26	105 ± 13	108 ± 25	0.002*^a,c^
		PS	137 ± 22	115 ± 15	113 ± 21	0.014*^a,c^
		PK	130 ± 14	119 ± 18	113 ± 27	0.121
	*p*		0.584	0.088	0.995	N/A
Propofol requirements (mg/kg)		PF		5.0 ± 0.94		<0.001†
		PS		3.8 ± 0.72		
		PK		2.4 ± 0.52		
	*p*					

Anesthesia was induced with intravenous (IV) propofol 8 mg/kg/min (propofol fast; group PF, *n* = 8) or propofol 2 mg/kg/min (slow; group PS; *n* = 8) or a single bolus of S-ketamine (2 mg/kg) and propofol 2 mg/kg/min (propofol–ketamine; group PK; *n* = 7). Data are presented as either mean ± standard deviation or median (interquartile range).

HR, heart rate; Propofol requirement, dose of propofol required to reach end-point of administration (mg/kg); BP, non-invasive blood pressure. †Significant differences between groups (*p* < 0.05). *Significant differences between time points (*p* < 0.05). aSignificant differences between baseline x T0; bSignificant differences between T0 x T15; cSignificant differences between baseline x T15.

## 5 Discussion

This study showed that, when within-group comparisons were made, the administration of propofol at fast rate or in combination with S-ketamine produced significant changes in some echocardiography variables of left ventricular function in healthy male cats (e.g., LVIDd, FS%, CO, and HR), which was not statistically significant with the propofol slow rate group. However, comparisons among treatments were not significantly different at any time point, which may show that changes observed in this study could have been due to individual variations within each group. In other words, it is not known if significant changes in echocardiography variables were truly of clinical relevance related to treatment groups or a result of within-group individual responses to treatment.

The amount of propofol required to achieve a defined end-point (T0) differed significantly between the three groups. Particularly, propofol requirements in PF (5.0 ± 0.9 mg/kg) were greater than PS (3.8 ± 0.7 mg/kg), as previously shown by Bauquier et al. (2017) (24) and Raillard & Murison (2018) (25), in cats and dogs, respectively. Such findings confirm that the rate of administration is important to decrease propofol requirements and likely its drug-related adverse effects, even when targeting the same end-point. The main reason for this higher propofol requirement in PF is that the time of administration is shorter than the time to reach equilibrium between plasma and cerebral concentrations (8). As a consequence, there is a delay in clinical effects of anesthesia, with an impact on propofol-administered doses at T0. Although co-administration of S-ketamine showed the lowest requirement of propofol doses (2.4 ± 0.5 mg/kg) of all treatments, as previously shown by Ilkiw et al. (2003) (9), this dose sparing effect was not enough to blunt changes in echocardiography variables. There was no difference in the total amount requirement of propofol infusion throughout the study between PF, PS, and PK protocols (2.0 ± 0.3 mL; 2.2 ± 0.8 mL; and 2.1 ± 0.5 mL, respectively).

End-diastolic volume is determined by preload, compliance, and diastolic filling time (12). Preload is associated with the left ventricular wall stress at the end of diastole and the pressure–volume relationship (12). Our findings demonstrate that LVIDd did not significantly change over time with fast or slow administration of propofol. However, an increase in LVIDd was observed when propofol was combined with S-ketamine. In brief, the relationship between HR, diastolic filling time, and end-diastolic volume is linked and can vary based on different factors (12). For example, a decrease in HR potentially increases diastolic filling time and end-diastolic volume, which could explain some of our findings (12). Ketamine is reported to have sympathomimetic effects, resulting in increased HR, CO, central venous pressure, and median blood pressure (26). However, hemodynamic depression can occur after the administration of ketamine in animals with reduced sympathetic tone (27). Of note, adrenoreceptor blockade can counteract the indirect sympathomimetic effects of ketamine. Evidence *in vitro* suggests that the positive inotropic effects of ketamine are mediated by the activation of cardiac β-adrenergic receptors, and ketamine seems to have a depressor effect on denervated cardiac muscle (28). According to Ward et al. (2012) (14), a mild decrease in LVIDd was reported (14) in cats sedated with acepromazine (0.1 mg/kg), in combination with butorphanol (0.25 mg/kg) either with or without ketamine (1.5 mg/kg). The findings of the present study indicated that observed changes using propofol in combination with S-ketamine could be due to a non-specific adrenoreceptor blockade potentially caused by premedication with acepromazine and/or reduced sympathetic tone caused by gabapentin, as shown by Allen et al. (2021) (29). Acepromazine antagonizes α-1 adrenoreceptors, leading to decreased peripheral vascular resistance, hypotension, and hypothermia even without producing significant changes in echocardiographic measurements in cats (30). Cats in all groups in our study received gabapentin–acepromazine–butorphanol; however, owing to the study design, it is not possible to isolate the specific effects of each of these drugs as interactions are likely.

Fractional shortening (FS%) is a widely used echocardiographic measurement for assessment of left ventricular function (31). It is a surrogate of systolic function and not a measure of contractility (32). Radial contractility, preload, afterload (32), and HR contribute to FS% (31). The slow propofol infusion did not produce statistically significant changes in FS%. A statistically significant reduction in FS% was observed at T0 in PF (47 to 34%) and PK (42 to 36%) but not in PS (45 to 37%). The PS had a higher effect on FS% than PK; however, the statistical test failed to reject the null hypothesis. This effect could be due to a reduced ventricular end-diastolic volume resulting from decreased systemic vascular resistance (SVR) caused by propofol, which would result in decreased preload and, consequently, reduced FS%. Ventricular function may also be assessed by EF%, which represents the fractional difference between end-diastolic and end-systolic volumes, i.e., the fractional stroke volume. As for FS%, EF% is highly dependent on contractility, preload, and afterload. In our study, EF% remained within normal range and neither change over time nor between the groups. These findings suggest reasonable stability in stroke volume determinants. Of note, if a reduction in venous return decreases BP and there is no perceived decrease in end-systolic volume, as observed in both propofol groups, a negative inotropic effect is likely to be present; however, this cannot be confirmed without further investigation (33). Analysis of myocardial strain using speckle tracking echocardiography could potentially detect local myocardial impairment (34) and should be considered in future studies for further investigations of the negative inotropic effect of propofol.

Non-invasive blood pressure was statistically reduced with both fast and slow rates of propofol but not when the drug was co-administered with S-ketamine. Thus, a slightly decrease of BP in PK was still observed. This contrasts with a study in which cats under propofol–ketamine anesthesia exhibited an improvement in mean blood pressure while other cardiovascular variables remain unchanged (9). The sympathomimetic effect of ketamine increases peripheral vascular resistance (9), theoretically counteracting the vasodilatory effects of propofol and its impact on BP. Regardless of the infusion rate of propofol, the transitory reduction in BP observed in this study suggest that propofol reduces LV preload by vasodilation. In dogs, systemic vascular resistance decreased immediately after anesthesia induction with propofol–ketamine, and the mean blood pressure remains stable (35). Studies suggest that propofol blocks Ca^2+^ channels, further inducing endothelial nitric oxide release and activation of protein kinase C (36). Despite the reduction in blood pressure, the slow administration of propofol did not cause BP to decrease below 90 mmHg in cats (23).

Cardiac output is a fundamental part of cardiac performance and ventricular function (20). Since CO involves the product of HR and stroke volume, it can be influenced by inotropism, lusitropy, preload, and afterload. Although thermodilution is considered the gold standard for obtaining CO, Doppler evaluation of pulmonary flow has been validated in propofol-anesthetized dogs (20). Despite the reduction in CO observed in fast administration of propofol at T15, it was only statistically significant when S-ketamine was used in combination with propofol. Of note, within-group comparisons were observed but not between groups. Thus, the possibility of a type II error might be attributed to an unpowered sample. In addition, this technique is not validated in cats which decreases reliability as it does not have reference values. In contrast with FS% changes which were transient, decreases in HR were observed until T15. Pronounced bradycardia (< 100 bpm) was observed after fast administration of propofol and even when the drug was given in combination with S-ketamine; however, there was marked HR variability between the protocols in this study that might have interfered with the results. Indeed, it is somehow surprising that S-ketamine did not increase HR and prevent bradycardia due to sympathetic stimulation. Bradycardia can be observed after the administration of propofol as the drug decreases preload mainly due to sympathetic inhibition and blunts baroreceptor activity and physiological compensatory mechanisms including reflex tachycardia (3, 10). Although some significant differences were identified within groups and individual variability was observed in some cats presenting high sympathetic tone, it is imperative to assess the clinical relevance of these findings, as most of them were transitory and likely not detrimental for this study population. However, these effects could have been relevant in cats with comorbidities and/or dehydration and hypovolemia during procedural sedation. Additionally, other factors may influence HR (and the cardiovascular system), such as autonomic tone, circulating volume, temperature, and age (37).

Gabapentin, an analog of the neurotransmitter γ-aminobutyric acid (GABA), is recommended by the International Society of Feline Medicine and the American Association of Feline Practitioners prior veterinary interactions, to reduce fear–anxiety responses in emotionally challenged cats (17). Catecholamine released due to stress can lead to vasoconstriction and increase sympathetic tone increasing propofol requirements (38). In this study, excessive manipulation and measurements of baseline echocardiographic values before premedication were avoided, and gabapentin was administered. The use of gabapentin before handling reduces fear- and aggressive-like behaviors while enhancing compliance scores (39). The drug reduces sympathetic tone (with reduced FS%), but systolic parameters remain within the normal reference ranges (29). The authors used gabapentin to reduce fear-induced behavior while preventing greater variations of catecholamine release during handling.

This study has limitations. The sample size was small, and a crossover trial could not be performed as cats were part of a spay-neuter program. Baseline echocardiography before gabapentin would have been relevant to the study; however, this was precluded by potential additional stress induced by handling. The inclusion of sedation scores would have been beneficial to discriminate the effects of stress on the hemodynamic variables. Non-invasive blood pressure was monitored using Doppler ultrasound. In this case, values for systolic blood pressure may not be accurate and may be lower than values obtained using invasive techniques (40). Cats were not intubated as this is not common practice during procedural sedation. However, propofol blunts laryngeal and pharyngeal reflexes; it is possible that regurgitation with aspiration may occur when the airways are not protected, and this is usually one of the main criticisms with the use of “top-up” doses of propofol during procedural sedation. Oxygen therapy was provided to prevent hypoxemia caused by hypoventilation, but this was never confirmed with arterial blood gasses. Hypoxemia is unlikely with oxygen supplementation via a tight face mask. For example, cyanosis was neither observed nor clear sympathetic responses to desaturation. The lack of end-tidal carbon dioxide monitoring is an important limitation in this study and cannot exclude the presence of hypoventilation and hypercapnia with an ultimate effect on echocardiography findings. Propofol produces dose-dependent respiratory depression by blunting hypoxic ventilatory responses and reducing tidal volume, minute volume, and respiratory rate (7). For this reason, when peripheral oxygen saturation is reduced, basic anesthetic monitoring, ventilatory support, and oxygen therapy are important to mitigate propofol-induced adverse effects on the clinical setting. Of note, propofol infusion was maintained after T0, thereby potentially altering T15 readings due to propofol redistribution and maintenance of plasma concentrations. Finally, the study involved specific and defined protocols of drug administration using infusion rates in healthy cats. It is not known how echocardiographic variables would change with boluses of propofol of unknown rates using different dosage and drug (including replacing S-ketamine with racemic ketamine) regimens, end points, or populations of cats with comorbidities and of different age and sex. The lack of oxygenation could also affect outcomes.

The fast rate of administration increased propofol requirements and affected transient variables of left ventricular systolic function in healthy male cats. Both fast rate and slow rate administration of propofol with S-ketamine induced changes in variables related to ventricular function. These within-group changes were not observed with the slow rate of administration of propofol alone, suggesting a potential benefit with the “slow and steady” approach during procedural sedation. Some of these changes in echocardiographic variables were still within normal reference values, and all protocols were well tolerated in healthy cats. The addition of S-ketamine seemed to prevent decreases in BP, but not bradycardia or depression of ventricular function in cats receiving slow rate administration of propofol.

## Data availability statement

The raw data supporting the conclusions of this article will be made available by the authors, without undue reservation.

## Ethics statement

The animal studies were approved by the Ethics Committee of the Veterinary Teaching Hospital of the Federal University of Parana (UFPR, Curitiba, Brazil, No. 024/021). The studies were conducted in accordance with the local legislation and institutional requirements. Written informed consent was obtained from the owners for the participation of their animals in this study.

## Author contributions

SM: Conceptualization, Data curation, Formal analysis, Investigation, Methodology, Resources, Visualization, Writing – original draft, Writing – review & editing. Data curation. MU: Data curation, Investigation, Software, Writing – review & editing. FA: Investigation, Writing – review & editing. LJ: Investigation, Writing – review & editing. JV: Investigation, Writing – review & editing. PS: Writing – review & editing. RV: Conceptualization, Data curation, Investigation, Methodology, Project administration, Resources, Supervision, Validation, Writing – review & editing.

## Footnotes

^1^ http://www.consort-statement.org

^2^ https://g-power.apponic.com

^3^ http://www.random.org
